# Electrospun cellulose acetate/gelatin nanofibrous wound dressing containing berberine for diabetic foot ulcer healing: *in vitro* and *in vivo* studies

**DOI:** 10.1038/s41598-020-65268-7

**Published:** 2020-05-20

**Authors:** Hadi Samadian, Sina Zamiri, Arian Ehterami, Saeed Farzamfar, Ahmad Vaez, Hossein Khastar, Mostafa Alam, Armin Ai, Hossein Derakhshankhah, Zahra Allahyari, Arash Goodarzi, Majid Salehi

**Affiliations:** 1 Pharmaceutical Sciences Research Center, Health Institute, Kermanshah University of Medical Sciences, Kermanshah, Iran; 2 Department of Kinesiology and Health Science, York University, Ontario, Canada; 3 Department of Mechanical Engineering, Science and Research Branch, Islamic Azad University, Tehran, Iran; 4 Department of Tissue Engineering and Applied Cell Sciences, School of Advanced Technologies in Medicine, Tehran University of Medical Sciences, Tehran, Iran; 5 Department of Tissue Engineering and Applied Cell Sciences, School of Advanced Medical Sciences and Technologies, Shiraz University of Medical Sciences, Shiraz, Iran; 6 School of Medicine, Shahroud University of Medical Sciences, Shahroud, Iran; 7 Department of Oral and Maxillofacial Surgery, Dental School, Shahid Beheshti University of Medical sciences, Tehran, Iran; 8 Dental student of scientific research center, faculty of dentistry, Tehran university of medical sciences, Tehran, Iran; 9 Department of Biomedical Engineering, Rochester Institute of Technology, Rochester, USA; 10 Department of Microsystems Engineering, Rochester Institute of Technology, Rochester, NY USA; 11 Department of Tissue Engineering, School of Advanced Technologies, Fasa University of Medical Sciences, Fasa, Iran; 12 Department of Tissue Engineering, School of Medicine, Shahroud University of Medical Sciences, Shahroud, Iran; 13 Tissue Engineering and stem cells research center, Shahroud University of Medical Sciences, Shahroud, Iran

**Keywords:** Nanomedicine, Nanoscale materials, Antimicrobials

## Abstract

Functional wound dressing with tailored physicochemical and biological properties is vital for diabetic foot ulcer (DFU) treatment. Our main objective in the current study was to fabricate Cellulose Acetate/Gelatin (CA/Gel) electrospun mat loaded with berberine (Beri) as the DFU-specific wound dressing. The wound healing efficacy of the fabricated dressings was evaluated in streptozotocin-induced diabetic rats. The results demonstrated an average nanofiber diameter of 502 ± 150 nm, and the tensile strength, contact angle, porosity, water vapor permeability and water uptake ratio of CA/Gel nanofibers were around 2.83 ± 0.08 MPa, 58.07 ± 2.35°, 78.17 ± 1.04%, 11.23 ± 1.05 mg/cm^2^/hr, and 12.78 ± 0.32%, respectively, while these values for CA/Gel/Beri nanofibers were 2.69 ± 0.05 MPa, 56.93 ± 1°, 76.17 ± 0.76%, 10.17 ± 0.21 mg/cm^2^/hr, and 14.37 ± 0.42%, respectively. The antibacterial evaluations demonstrated that the dressings exhibited potent antibacterial activity. The collagen density of 88.8 ± 6.7% and the angiogenesis score of 19.8 ± 3.8 obtained in the animal studies indicate a proper wound healing. These findings implied that the incorporation of berberine did not compromise the physical properties of dressing, while improving the biological activities. In conclusion, our results indicated that the prepared mat is a proper wound dressing for DFU management and treatment.

## Introduction

Diabetes mellitus is classified as a metabolic disease that has various complications such as chronic wounds, arterial damage, and neuropathy resulting from uncontrolled blood sugar. The wound healing process is a complex and multiphase process that is delayed in diabetic patients because of various complexities^1,2^. In these patients, the angiogenesis and re-epithelialization are inadequate because of low interaction between growth factors and their target site. Severe inflammation is an additional deleterious factor resulting from neutrophil infiltration. Moreover, diabetic foot ulcer (DFU) is another complication that is the consequence of intense inflammation, limited nutrients, and poor blood circulation. Despite the tremendous breakthroughs over the last decades, effective treatment of DFU remains a challenge^3–5^.

Since DFU is an acute wound, it needs to be dressed with proper dressing materials able to enhance the healing process and also isolate the wound site from pathogen microorganisms^6,7^. Moreover, the wound dressing must be able to absorb the exerted exudates from the wound and also provide an optimum moist environment to expedite the healing process. Besides, the healing process of DFU requires to be accelerated via bioactive molecules or drugs, and the proposed dressing must be able to load a proper amount of the drug and release it in a sustain release manner^8–10^. A wide range of biomaterials and nanostructured materials have been evaluated as wound dressings for DFU, such as natural and synthetic polymers in the forms of hydrocolloids, hydrogels, foams, and electrospun nanofiber dressing^11,12^.

Among the viable candidates, electrospun nanofibrous mats offer a wide range of promising possibilities suitable for wound dressing applications. Electrospinning is a sophisticated and efficient technique which provides a low-cost, scalable, flexible, relatively simple approach for nanofibers fabrication from a wide variety of synthetic and natural substance^13–16^. The porosity and the pore size of the electrospun mats can be adjusted to inhibit microorganism penetration, while oxygen can easily pass through the dressing and reach the wound site. Interestingly, the water vapor transmission can be tailored to provide the ideal moisture condition for the wound healing process. The high surface area of nanofibers is favorable for drug loading and sustained delivery. The intended drugs, natural substance, or bioactive molecules can be adsorbed onto the surface of nanofibers or encapsulated into the nanofibers matrix^17,18^. Moreover, the electrospun nanofibrous dressings are self-standing and their handling during the wound treatment is easy^19–21^.

Electrospun nanofibers have been fabricated from a variety of natural and synthetic polymers and applied as the wound dressing. Among them, natural polymers have grabbed considerable attention due to their desirable properties. Most of them are biocompatible, non-toxic, biodegradable, abundant, inexpensive, renewable, and versatile^22–24^. Cellulose is one of the most abundant natural polymers on earth, which has various derivatives. Still, the most promising derivative is cellulose acetate (CA), the acetate ester form of cellulose^25,26^. CA is applicable in various applications such as membrane separation, biomedical, textile fibers, cigarette industries, and plastics^27–30^. The biomedical applications of CA are mainly categorized in drug delivery systems, tissue engineering, and wound dressing^31–34^. Biocompatibility, water absorption abilities, and good interaction with fibroblast cells have mad CA the right candidate for wound dressing applications^35^. Gelatin (Gel) is a widely used natural polymer with fascinating biological properties such as biocompatibility, biodegradability, and bioactivity, which is obtained from collagen hydrolysis^36–38^. Various forms of gelatin can be fabricated, such as the hydrogel, layered, freeze-dried, and micro- and nanofibers for different applications. Due to the presence of cell adhesion domains such as the Arg-Gly-Asp (RGD) domains in the structure of gelatin, it is widely used in tissue engineering and wound dressing applications^39–41^.

In addition to the structural requirements, a proper DFU dressing should have an active ingredient to either enhance the healing process or even provides the antibacterial property. Various types of biological, natural, and chemical moieties have used to induce biological functions to the dressings. Berberine is a natural substance belongs to the alkaloid family found in the rhizome, roots, and stems of various plants such as Oregon grape, Goldenseal, and Barberry^42^. Berberine is known for its anti-diabetic, antimicrobial, and anti-inflammatory activities^43^. Moreover, some studies reported the diabetic wound healing efficacy of berberine^44^. Accordingly, the main objective of our study is to fabricate CA/Gel electrospun nanofibrous mat containing berberine as a DFU wound dressing.

## Results and Discussion

### The morphology of nanofibers

**Figure 1:**
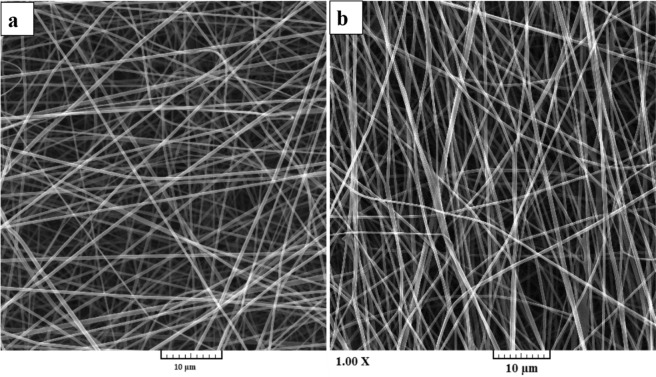
SEM images of the electrospun (**a**) CA/Gel and (**b**) CA/Gel/Beri nanofibers.

Various characterization methods were used to assess the properties of the fabricated nanofibers. The morphology of the prepared nanofibers was observed by using SEM imaging (Fig. 1). The SEM images showed that the fabricated CA/Gel and CA/Gel/Beri nanofibers are uniform and straight without any beads and deformities. It is apparent that, the incorporation of berberine had no adverse effect on the morphology of nanofibers. The image analysis using ImageJ software (U. S. National Institutes of Health, Bethesda, Maryland, USA) showed that the diameter of CA/Gel and CA/Gel/Beri nanofibers were 425 ± 79 and 502 ± 150 nm, respectively.

Vatankhah *et al*.^45^ fabricated CA/Gel nanofibers as the wound dressing materials. They combined CA and Gel with different weight ratios (75:25, 50:50, and 25:75 (wt.%)) and reported the nanofibers diameters of 260 ± 105, 227 ± 92, and 198 ± 52 nm, respectively. They observed the highest human dermal fibroblasts proliferation on the Ca/Gel 25:75 nanofibers. In another study, Kusumah *et al*.^46^ reported the fabrication of smooth and beadless Ca/Gel nanofibers with a diameter of 649 ± 21 nm. Obtained nanofibers with different diameters in various studies can be related to the different applied electrospinning parameters, such as applied voltage, nozzle to collector distance, and feeding rate.

### Mechanical property

The mechanical properties of the nanofibers were measured by the tensile strength method based on ISO 5270:1999 standard test methods. The results showed that the incorporation of berberine compromised the mechanical property and slightly reduced the tensile strength from 2.83 ± 0.08 to 2.69 ± 0.05 MPa; however, the observed difference was not statistically significant (p < 0.32). Our previous study^47^ showed that the addition of taurine to electrospun Poly (ɛ-caprolactone)/Gelatin nanofibers reduced the mechanical property. The obtained results can be attributed to the berberine-induced reduction in the physical interaction between the polymer chains. Berberine weakened interchains and intrachain physical interaction and forces such as van der Waals forces and hydrogen bonding^47,48^. However, the obtained tensile strengths are in the acceptable range. Vatankhah *et al*.^45^ reported the tensile strength in the range of 15 to 3 Mpa for CA/Gel nanofibers fabricated from different weight ratios of CA and Gel. In another study, Waghmare *et al*.^49^ fabricated starch-based nanofibrous wound dressing and reported the tensile strength in the range of 0.57–0.88 MPa for the produced nanofibers. Moreover, it is reported that the tensile strength ranging from 0.7 to 18.0 MPa is sufficient for dermal cell culture^49–51^.

### Porosity

The liquid displacement technique was used to measure the porosity of the fabricated nanofibers. The results showed that the porosity of CA/Gel and CA/Gel/Beri nanofibers were 78.17 ± 1.04 and 76.17 ± 0.76%, respectively. Chong *et al*.^52^ concluded that the porosity in the range of 60–90% is preferred for tissue engineering applications. They also reported the porosity range of 60–70% for their fabricated electrospun PCL/Gel nanofibrous scaffolds. In another study, Shan *et al*.^53^ reported a porosity value of around 87% for the fabricated silk fibroin/Gel electrospun nanofibrous dressing. Although the porosity of the fabricated CA/Gel/Beri nanofibers are in the acceptable range, Water vapor permeability (WVP) should be measured along to conclude the efficacy of the prepared CA/Gel/Beri nanofibers.

### Wettability

A suitable dressing should be able to absorb the wound exudates and maintain the moisture of the wound site. These criteria are under the influence of the surface wettability and hydrophilicity of the dressing. The wettability of the fabricated dressings was measured based on the water contact angle method, and the results are presented in Table 1. The water contact angle of CA/Gel and CA/Gel/Beri nanofibers were 58.07 ± 2.35° and 56.93 ± 1°, respectively, which indicated that the fabricated dressings are hydrophilic and proper for absorbing exudates and maintaining the moisture of the wound bed. Liu *et al*.^54^ demonstrated that the incorporation of Gel could increase the surface wettability of cellulose acetate due to its hydrophilic nature.

**Table 1:** Characteristic of the fabricated CA/Gel and CA/Gel/Beri nanofibers.

Samples	Tensile strength (MPa)	Porosity (%)	Contact angle (°)	WVP mg/cm^2^/hr	Water uptake ratio (%)	Weight loss (%)	
						Day 7	Day 14
CA/Gel/0% beri	2.83 ± 0.08	78.17 ± 1.04	58.07 ± 2.35	11.23 ± 1.05*	12.78 ± 0.32	38.0 ± 3.0	74.0 ± 5.0
CA/Gel/1% beri	2.69 ± 0.05	76.17 ± 0.76	56.93 ± 1	10.17 ± 0.21*	14.37 ± 0.42	50.0 ± 3.0	79.0 ± 7.0
Open container	21.53 ± 0.42						

^*^p < 0.05.

Abbreviations: CA: Cellulose acetate, Gel: Gelatin, beri: Berberine, WVP: Water vapor permeation.

### Water vapor permeability and water-uptake capacity

The vapor exchange through the dressing is a critical property determining the efficacy of the dressing. High WVP value dehydrates the wound and induces scar formation, while the low WVP value delays the wound healing process due to the deposited exudates. Therefore, a proper dressing should exhibit an optimum value of WVP. The results showed that the WVP value for CA/Gel dressing was 11.23 ± 1.05 mg/cm^2^/hr, while the addition of berberine reduced this value to 10.17 ± 0.21 mg/cm^2^/hr which both values are significantly lower than the control group (the open container) (p < 0.05).

As shown in Table 1, the water uptake ratio of CA/Gel was 12.78 ± 0.32%, and the incorporation of berberine increased the ratio to 14.37 ± 0.42%. This enhancement in the water uptake ratio can be related to the hydrophilic nature of berberine. These results demonstrate that the fabricated CA/Gel and CA/Gel/Beri can properly absorb the wound exudates and subsequently improve the wound healing process.

### Weight loss assay findings

The degradation rate of the prepared CA/Gel and CA/Gel/Beri was measured in PBS at days 7 and 14 (Table 1). The results indicated that the fabricated dressings undergo significant weight loss despite remaining structurally stable during 14 days, and the highest weight loss, around 80%, was observed in CA/Gel/Beri group on days 14.

As shown in Table 1, the incorporation of berberine accelerated the weight loss of CA/Gel nanofibers at both time intervals, which was statistically significant at 7 days (p < 0.05). The observed increased weight loss can be related to the hydrophilic nature of berberine, which enhanced the interactions between CA/Gel nanofibers and water molecules. Moreover, berberine may reduce the physical interactions between the polymer chains, which facilitates the degradation rate. However, in the wound dressing applications, there is no need for biodegradability.

### Microbial evaluations findings

#### Microbial penetration

A proper wound dressing must withstand against microbial invasion through the dressing and show acceptable microbial barrier property. In this experiment, the negative control was the tube closed with a cotton ball to test the sterilization procedure, while the positive control was open tube and tested to ensure the growth possibility of bacteria in the nutrient broth.

**Figure 2:**
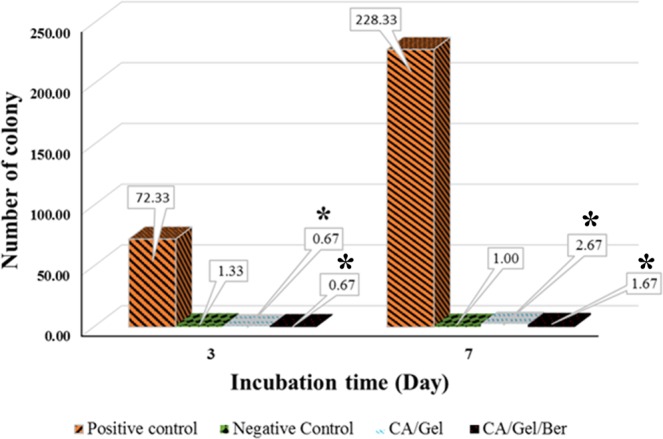
Microbial barrier property of the fabricated dressing after 3 and 7 days incubation, measured by colony counting assay. Values represent the mean ± SD, n = 3. *p < 0.05 in comparison with the positive control group (obtained by one-way ANOVA).

**Figure 3:**
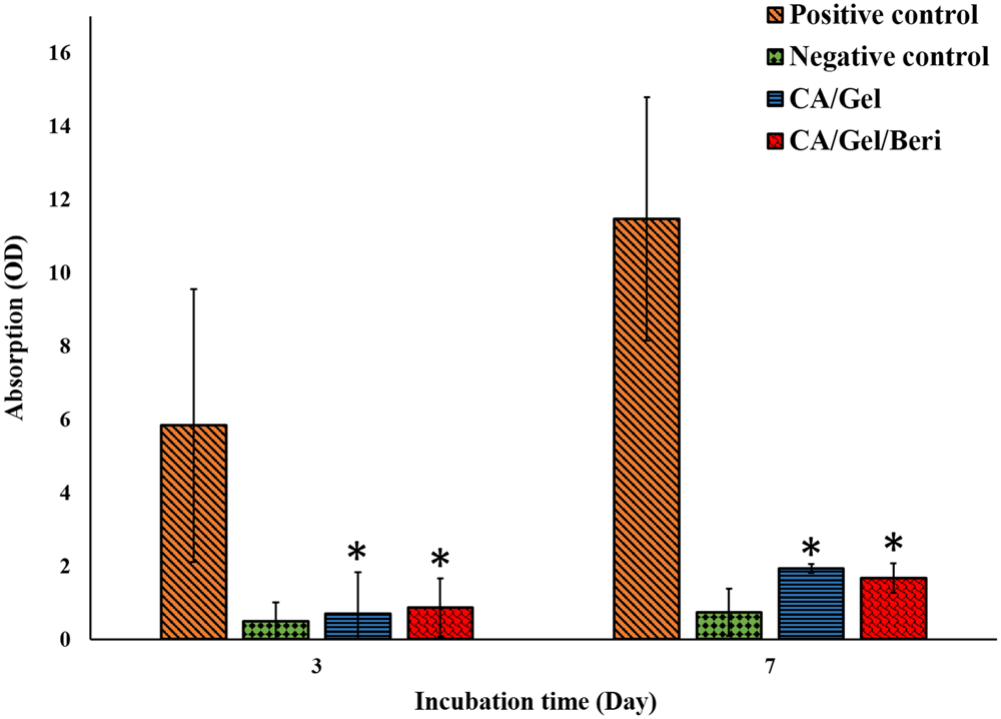
Microbial barrier property of the fabricated dressing after 3 and 7 days incubation measured by Spectrophotometer at 600 nm. Values represent the mean ± SD, n = 3. *p < 0.05 in comparison with the positive control group (obtained by one-way ANOVA).

As shown in Fig. 2, the fabricated dressings prevent bacterial transition, and negligible colonies were grown in the culture media, which was statistically significant compared with the positive control (P < 0.005). Moreover, the cloudiness of the nutrient broth was further evaluated by Spectrophotometer at 600 nm, and the results are shown in Fig. 3.

The turbidimetry assay results confirmed the colony counting assay findings, which indicated that the fabricated dressings exhibited excellent microbial barrier property. The results demonstrated that the microbial penetration of CA/Gel/Beri dressing was lower than CA/Gel (p < 0.23), which can be related to the antibacterial property of berberine. The antibacterial activity of berberine was shown by Peng *et al*.^55^. Moreover, it is shown that even 64 layers of gauze were not able to stand against bacterial penetration into the wound^56^. Hence, these results imply that the fabricated dressings are suitable for wound care applications.

#### Antibacterial assay findings

An effective wound dressing should possess the antibacterial activity along with bacterial penetration barrier. The antibacterial activities of the fabricated dressings were assessed by time-kill assay against gram-positive and gram-negative bacterium *Staphylococcus aureus* and *Pseudomonas aeruginosa*, respectively (Table 2).

**Table 2:** The antibacterial activities of the dressings evaluated by the time-kill assay (number of colony-forming units).

Dressings	Minimum Inhibition Concentrations (MIC)							
	S.U-1h	S.U-2h	S.U-4h	S.U-24h	PS.a-1h	PS.a-2h	PS.a-4h	PS.a-24h
Positive control	22 ± 1.2	87 ± 5.5	201 ± 4.9	552 ± 9.1	21 ± 1.8	39 ± 3.1	83 ± 5.01	493 ± 6.6
CA/Gel	20 ± 2.5	72 ± 3.6	193 ± 7.16	499 ± 6.3	18 ± 0.2	33 ± 2.3	76 ± 4.7	460 ± 6.1
CA/Gel/Beri	17 ± 1.4	50 ± 2.9	78 ± 4.9	70 ± 3.7	16 ± 3.1	25 ± 3.06	31 ± 0.7	44 ± 5.7

Abbreviation: MIC: Minimum Inhibition Concentrations, S.U: *Staphylococcus aureus*, PS.a: *Pseudomonas aeruginosa*, CA: Cellulose acetate, Gel: Gelatin, beri: Berberine.

The results exhibited that the antibacterial activity of the fabricated CA/Gel/Beri dressing was significantly higher than the positive control and the CA/Gel dressing (p < 0.005). Previous studies confirmed the potential antibacterial activity of berberine against various bacteria^55,57,58^. Kang *et al*.^57^ reported that berberine exhibited time and concentration dependence antibacterial activity against *Actinobacillus pleuropneumoniae* with the MIC of 0.3125 mg/mL. Wojtyczka *et al*.^59^ reported that berberine has antibacterial activity against coagulase-negative staphylococcus strains with the MIC ranged from 16 to 512 μg/mL. The higher bactericidal effect of CA/Gel/Beri dressing observed in our study is related to the incorporation of berberine in the matrix of CA/Gel nanofibers, while this value is effective for wound dressing applications. These results imply that the fabricated dressings are not only a barrier against bacterial penetration but also an antibacterial dressing.

#### The hemocompatibility results

**Figure 4:**
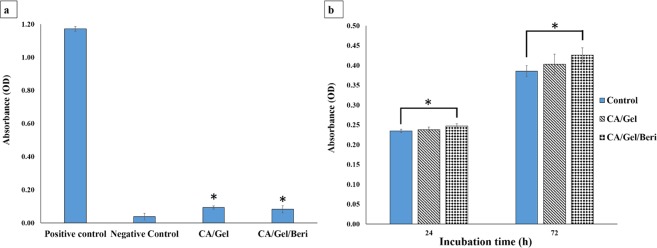
Biocompatibility histogram of the fabricated dressings. (**a**) Hemocompatibility histogram. (**b**) Cell proliferation assay histogram measured by MTT assay after 24 and 72 h cell seeding. Values represent the mean ± SD, n = 3, *p < 0.05 in comparison with the positive control group (obtained by one-way ANOVA).

Since wound dressings are designed to contact with bloody wounds, it is essential to assess the hemocompatibility of the fabricated dressings. The hemolysis induced by the fabricated dressing was measured as an indication of hemocompatibility, and the obtained results are presented in Fig. 4a. The obtained results from the hemocompatibility test showed that the hemolysis induced by the fabricated dressings were significantly lower than the positive control (distilled water-lysed RBC) (p < 0.05). Moreover, it is shown that the berberine incorporated dressing exhibited lower hemolysis than pure CA/Gel dressing. Vuddanda *et al*.^60^ synthesized berberine chloride nanoparticles and reported less than 10% hemolysis under incubation with human blood samples. They proposed that the electrostatic interaction between positively charged nanoparticle and negatively charged RBCs may have induced the observed hemolysis.

#### Cell proliferation assay results

The proliferation of the L929 murine fibroblastic cell line was measured by MTT assay at 24 and 72 h defter cell seeding, and the results are present in Fig. 4b. It is shown that the maximum cell growth was obtained with the CA/Gel/Beri dressing, which was statistically significant compared with the control group (tissue culture plate) (p < 0.005). It is also apparent that the incorporation of berberine enhanced the proliferation of the L929 murine fibroblastic cells. Liu *et al*.^61^ reported that berberine is able to promote the proliferation of periodontal ligament stem cells (hPDLSC) through the activation of the ERK-FOS signaling pathway via binding to the EGFR on the cell membrane.

#### The morphology of the cells on the nanofibers

**Figure 5:**
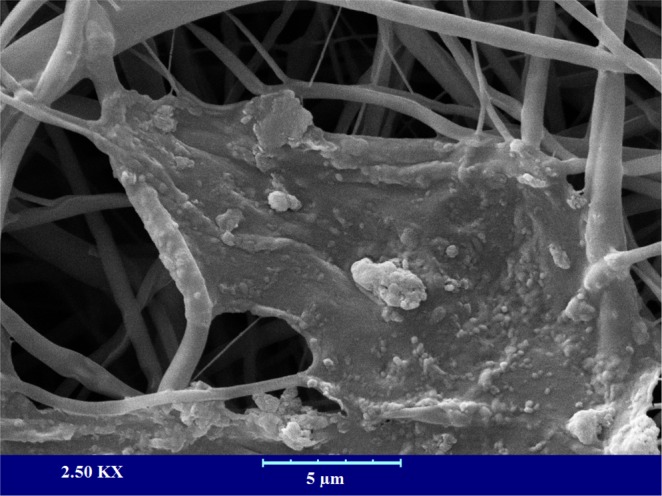
The SEM micrograph of the cultured L929 murine fibroblastic cell on nanofibers after 24 h cell seeding.

The morphology of the seeded L929 murine fibroblastic cell on the CA/Gel/Beri was observed using SEM after fixation and dehydration. As shown in Fig. 5, the cultured cells well spread onto the nanofibers after 24 h cell seeding. This image clearly depicts that the cells are attached and spread onto the fabricated nanofibers.

### Animal study findings

#### Histopathology and Histomorphometry analysis results

**Figure 6:**
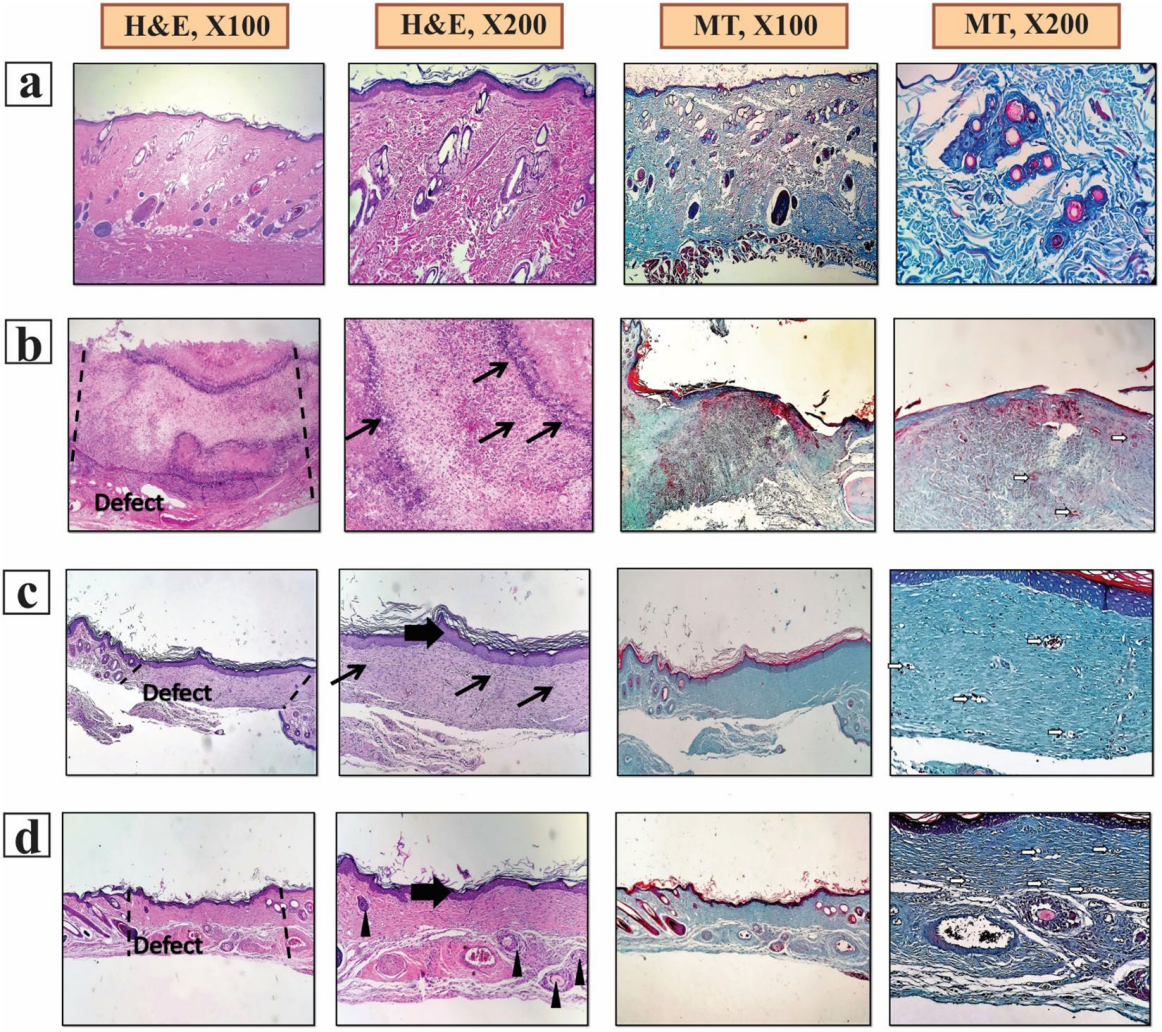
Haematoxylin and Eosin (H&E) and Masson’s trichrome (MT) stained microscopic sections of wounded tissue treated with dressings 16 days post-treatment. (**a**) The positive control, (**b**) the negative control, (**c**) the CA/Gel dressing, and (**d**) the CA/Gel/Beri dressing. Thick arrows: epidermal layer, thin arrows: infiltration of inflammatory cells, arrowheads rejuvenation of skin appendages, white arrows: neo-vascularization.

The histopathology examinations evaluated the wound healing efficacy of the fabricated CA/Gel and CA/Gel/Beri dressings, and the results are presented in Fig. 6. The obtained tissues were stained with H&E and MT stainings. Figure 6a shows the positive control (healthy skin) with intact dermal and epidermal, whereas the negative control group (untreated wound) exhibited polymorphonuclear inflammatory cells (PMNs) infiltration and granulation tissue formation; however, the epidermal layer has not been formed yet (Fig. 6b).

The CA/Gel dressing group showed the completed epithelialization and less infiltrated PMNs in comparison with the negative control group (Fig. 6c). The anti-inflammatory activity of berberine has been shown in other studies. Xiao *et al*.^62^ reported that berberine has an anti-inflammatory effect through the adjusting PCSK9-LDLR pathway and reducing the plasma concentration of IFNγ, TNFα, IL-1α, and 8-isoprostane. Yi *et al*.^63^ demonstrated that berberine acts as an antiatherosclerotic agent through the inhibition of COX-2 expression via the ERK1/2 signaling pathway. In another study, Kim *et al*.^64^ conducted a study to assess the anti-inflammatory effect of berberine on normal human keratinocytes. They reported that berberine treatment reduced IL-6 expression, activity, and expression of matrix metalloproteinase-9 (MMP-9). Moreover, they observed that berberine suppressed AP-1 DNA binding activity and ERK activation.

The epidermal proliferation and the epidermal layer enlargement were evident in CA/Gel/Beri group (Fig. 6d), while the inflammatory response and the granulation tissue decreased in this group. Rejuvenation of skin appendix was seen and developed in CA/Gel/Beri group. Moreover, this group exhibited more resemblance to healthy skin with a thin epidermis, the presence of normal rete ridges, and a standard thickness of skin layers. The histopathological observations showed that the fabricated CA/Gel/Beri dressing resulted in the best outcome compared to negative control and CA/Gel groups.

The histomorphometric analysis was done to further evaluate the healing process of the wounded skin (Table 3). The minimum re-epithelialization was observed in the negative control group, while the CA/Gel/Beri exhibited the highest re-epithelialization after 16 days (P < 0.05). The wound site in the negative control group was filled with immature granulation tissue, which indicated the slow and ineffective wound healing process.

**Table 3:** Histomorphometric analysis of different experimental groups.

Groups	Collagen density (%)	Angiogenesis score	Epitheliogenesis Score (n = 4)
Negative control	29.5 ± 2.3	9.3 ± 1.2	0,0,1,0
CA/Gel	71.7 ± 5.2 **	12.1 ± 2.7	3,2,2,3 *
CA/Gel/Beri	88.8 ± 6.7***	19.8 ± 3.8**	4,4,4,4 ***

Values indicates treatment group versus un-treatment group (empty control); *P < 0.05, **P < 0.01, ***P < 0.001.

Abbreviations: CA: Cellulose acetate, Gel: Gelatin, beri: Berberine, WVP: Water vapor permeation.

The wound healing process is critically dependent on the angiogenesis, as well as the collagen synthesis in the wound site. In this regard, the angiogenesis and collagen synthesis were measured to further assess the wound healing process under the influence of the fabricated dressings. The highest collagen density, 88.8 ± 6.7%, was observed in CA/Gel/Beri, which was statistically significant compared with the CA/Gel, 71.7 ± 5.2%, and the negative control group, 29.5 ± 2.3%, (P < 0.01 and P < 0.001). Moreover, CA/Gel/Beri exhibited the highest angiogenesis, 19.8 ± 3.8, while the lowest angiogenesis, 9.3 ± 1.2, was observed in the negative control group (P < 0.001). Previous studies demonstrated the wound healing potential of berberine treatment. Pashaee *et al*.^44^ evaluated the wound healing activity of berberine on STZ-induced diabetic rats and conducted that the treatment improved the wound healing process. They proposed that the observed healing process is related to the anti-diabetic, anti-inflammatory, as well as antimicrobial effects of berberine. Our findings indicated that the incorporation of berberine into the dressing significantly improved the healing process.

## Conclusion

DFU is one of the main disabling complications of Mellitus diabetes, which requires excessive consideration and wound care management. The healing process in DFU is delayed and takes a long time to heal, so accelerating agents should be applied to enhance the healing process. Moreover, the wound site must be isolated from pathogenic microorganisms via a well-designed dressing. Electrospun nanofibrous dressings are ideal wound dressing due to their nanometric scale, high surface to volume ratio, adjustable porosity, and ability to load various drugs and bioactive molecules. In the present study, CA/Gel nanofibrous dressing containing berberine was fabricated using the electrospinning technique and after characterization used as a DFU dressing material. The characterization results demonstrated that the fabricated dressing was suitable as the wound dressing material and did not induced any adverse effect on the cultured cells and also exhibited antibacterial activity against gram-positive and gram-negative bacterium. The animal studies on the STZ-induced diabetic rats demonstrated that the CA/Gel/Beri dressing enhanced the wound healing process. The evidence from this study suggests that the fabricated CA/Gel/Beri dressing is suitable dressing material to enhance the healing process of DFU and also isolate the wound site from the pathogenic microorganisms invasion.

## Materials and methods

### Chemicals

Gelatin (bovine skin, type B), CA [Mw = 30 kDa, acetyl content = 39.70% (w/w)], 1,1,1,3,3,3-hexafluoro-2-Propanol (HFP), berberine >90%, glutaraldehyde (GA) 25% in H_2_O, streptozotocin (STZ), ketamine, xylazine, and MTT assay kit were purchased from Sigma-Aldrich (St. Louis, MO). Brain heart infusion (BHI) broth culture medium obtained from (Darmstadt, Germany). Dulbecco’s modified Eagle’s medium: nutrient mixture F-12 (DMEM/F12), fetal bovine serum (FBS), penicillin and streptomycin were purchased from (Grand Island. NY). GOD-POD (glucose oxidase-peroxidase) diagnostic kit was obtained from Accurex Biomedical Pvt. Ltd., Mumbai, India. L929 murine fibroblastic cell line and adult Wistar rats were obtained from Pasteur Institute, Tehran, Iran.

### Fabrication of nanofibrous wound dressing

A proper amount of gelatin and CA were separately dissolved in HFP to obtain the final concentration of 6% (w/v). CA (6% (w/v)) and Gel (6% (w/v)) were combined at the weight ratio of 25:75 and stirred for 24 h. In the next step, the berberine was added to the resulted solution in the amounts of 1% (w/w)^65^ of the total polymer content and stirred for 24 h. The commercial electrospinning apparatus (NanoAzma, Tehran, Iran) was used to fabricate the electrospun wound dressing. Briefly, the CA/Gel/Beri solution was loaded into a 10 mL disposable syringe ending to a 25-gauge stainless steel blunted needle. The needle was connected to the power supply and the polymer solution was pumped onto the tip of the needle via a syringe pump at the constant rate. The applied voltage, the feeding rate, and the nozzle to the collector distance were 15 kV, 0.2 mL/h, and 15 cm, respectively. The cross-linking was conducted to prevent the dissolution of gelatin in the biological situation. The fabricated mats were incubated with the vapor of 10% (w/v) glutaraldehyde (GA) for 16 h to induce cross-linking between the nanofibers^66^. The cross-linked nanofibers then washed thoroughly by distilled water to remove unreacted GA followed by drying at room temperature.

### Characterization of the wound dressing

#### Scanning electron microscopy analysis

The produced wound dressings were sputter-coated with a thin layer of gold using a sputter coater (KYKY Technology Development, Beijing, China) and imaged using a scanning electron microscope (SEM; KYKY Technology Development, Beijing, China) at an accelerating voltage of 26 kV.

#### Mechanical strength measurement

The tensile strength of the fabricated wound dressings was measured by a uniaxial tensile testing device (Santam, Karaj, Iran) with an extension rate of 1 mm/min, according to ISO 5270:1999 standard test methods.

#### Contact angle measurement

The water contact angle was measured as the indication of hydrophilicity/hydrophobicity nature of the dressings via a static contact angle measuring device (KRUSS, Hamburg, Germany). The water droplet was poured onto the three different points of each sample, and the resulted angle between the water droplet and the surface of each specimen was averaged and reported.

#### Water vapor permeability (WVP) test

The flexible bottle permeation test (Systech, UK) was used to measure WVP of the fabricated dressings. The dressings-capped bottles were incubated at 33 °C for 12 hours, the evaporated water through the dressing was measured, and Eq. 1 was used to calculate WVP at steady-state.

1 $$\text{WVP}\,=\,\frac{\text{W}}{\text{AT}}$$

Where W, A, and T stand for the mass of water lost, area (1.18 cm^2^) of dressing, and exposure time, respectively.

#### Water uptake ratio evaluation

The water uptake ability of the mats was measured based on our previous studies^47,67^. The dry dressing was weighed (W_0_), immersed in distilled water at room temperature for 24 h, and then the wet samples were weighed again (W_1_). Equation 2 was used to calculate the water-uptake capacity of the dressings. Three measurements were conducted for each specimen and the averages were reported.

2 $$Water\,Uptake\,( \% )=\frac{\text{W}1-\text{W}0}{\text{W}0}\times 100$$

#### Porosity assessment

The porosity of the electrospun dressings was measured based on the liquid displacement technique using Eq. 3^68^. Briefly, the mat of weight W was immersed in a graduated cylinder containing a known volume (V1) of ethanol, and the resulted volume then was recorded as V2. After 10 min, the samples were removed, and the residual ethanol volume recorded as V3.

3 $$\text{Porosity}\,( \% )=\frac{\text{v}1-\text{v}3}{\text{v}2-\text{v}3}\times 100$$

#### Weight loss measurement

Weight loss of the dressings was measured as the function of degradation based on our previous study^67^. Briefly, a proper amount of dressing was weighted (W_0_), incubated in PBS solution, extracted for the solution after 7 and 14 days, and precisely weighted (W_1_). Equation 4 was used to calculate the weight loss.

4 $$\text{Weight}\,\text{loss}\,( \% )=\frac{\text{W}0-\text{W}1}{\text{W}0}\times 100$$

#### Microbial penetration test

The microbial penetration test assessed the resistance of the fabricated dressings against microbial penetration. Briefly, 10 ml vials (test area: 0.8 cm^2^) containing 5 ml of BHI broth culture medium was capped with the prepared mates, kept at ambient conditions, and growth of the bacteria into the culture medium was measured after 3 and 7 days. Bottles covered with the cotton ball and open vials served as negative and positive controls, respectively. The turbidity as the indication of microbial contamination was measured by spectroscopy approach at 600 nm using a microplate spectrophotometer (n = 3).

#### Antibacterial growth assay

The antibacterial activities of the prepared nanofibers were conducted based on the time-kill assay against *Staphylococcus aureus* and *Pseudomonas aeruginosa*^69^. Briefly, the prepared CA/Gel nanofibers were added to bacterial suspensions (previously adjusted to 1 × 10^7^ CFU/ml) at concentrations of 1/2-fold of the Minimum Inhibition Concentrations (MIC). 0.5 mL of each suspension was incubated at 37 °C with gentle agitation in a shaking water bath for 24 h. After the incubation period, the suspension (10 μL) was serially-diluted and inoculated on agar plates and incubated for 1, 2, 4, and 24 h in aerobic incubation condition at 37 °C. Then, the number of viable bacteria colonies was counted.

#### Blood compatibility or hemolysis assay

The hemolysis assay was conducted on human whole blood anticoagulated and diluted with normal saline. The samples were incubated with 200 µl of blood samples at 37 °C for 60 min, followed by centrifugation at 1500 rpm for 10 min. Then the absorbance of the resulted supernatant was read at 545 nm using a Multi-Mode Microplate Reader (BioTek Synergy 2). The negative control and positive control were the whole blood diluted in normal saline and the whole blood diluted in deionized water, respectively. Equation 5 was used to calculate the hemolysis percent^70^.

5 $$\text{Hemolysis}\, \% =\frac{Dt-Dnc}{Dpc-Dnc}\times 100 \% $$

Where Dt is the absorbance of the sample, Dnc is the absorbance of the negative control, and Dpc is the absorbance of the positive control.

#### Cell culture studies

L929 murine fibroblastic cell line was obtained from Pasteur Institute, Tehran, Iran, and used to evaluate the biocompatibility of the prepared dressing. DMEM/F12 enriched with 10% (v/v) FBS, 100 unit/mL of penicillin, and 100 µg/mL of streptomycin. The dressings were cut spherically and put into the bottom of each well of a 96-well plate followed by UV light exposure for one h for sterilization. Then, the nanofibrous mat was washed with PBS twice and once with DMEM/F12 and then seeded with 1 × 10^4^ cells. MTT assay kit was used to evaluate the proliferation of the seeded cells based on the previously described method^71^. The positive control was the wells without dressings, the experiment was done triplicated, and the average data reported.

The morphology of cultured cells in the fabricated nanofibers was observed using SEM imaging. Briefly, after 24 h, the seeded cells were fixed in 4% paraformaldehyde for one h at room temperature, dehydrated in graded ethanol, sputter-coated with a thin layer of gold, and observed at an accelerating voltage of 26 kV.

### Animal studies

#### Induction and assessment of diabetes

The animal studies were conducted on adult Wistar rats purchased from Pasteur Institute, Tehran, Iran. Diabetes was induced by intraperitoneal injection of a single dose of 55 mg/kg STZ in citrate buffer (pH 4.4, 0.1 M). The equal volume of citrate buffer was injected intraperitoneally to the age-matched control rats. The retro-orbital plexus technique was used to collect the blood samples, and GOD-POD (glucose oxidase-peroxidase) diagnostic kit was used to measure serum glucose levels, and the levels of more than 250 mg/dL confirmed the diabetes induction^72^.

#### *In vivo* wound healing study

The animal study was conducted on 24 male adult Wistar rats (Weight 200–230 g) based on the instruction of the ethics committee of Kermanshah University of Medical Sciences. General anesthesia was induced by intraperitoneal injection of Ketamine 5%/Xylazine 2% (70 mg ketamine and 6 mg Xylazine/1 kg body weight). The full-thickness excisional wound model was created on foot of each rat as a rectangular pattern marked on the dorsal surface of the foot using a flexible transparent plastic template, and then a layer of skin in full-thickness with a standard area of 2 mm × 5  m was removed. The animals were divided into 4 groups (6 rats per group), and the wounds were treated with the sterilized CA/Gel, CA/Gel/Beri nanofibers, and the sterile gauze as the negative control. An elastic adhesive bandage was applied to fix and secure the dressings on the wounded area and the dressings changed daily.

After 16 days post-surgery, the animals were sacrificed by ketamine overdose injection and the wound tissue was harvested and fixed in 10% buffered formalin. The harvested tissues were processed, embedded in paraffin, sectioned, and stained with hematoxylin-eosin (H&E) and Masson’s trichrome (MT). An independent pathologist observed and interpreted the prepared slides under a light microscope (Carl Zeiss, Thornwood, USA) with a digital camera (Olympus, Tokyo, Japan) and photographed at ×100, and ×200 magnification. Epithelialization, inflammatory cell infiltration, fibroplasia, and granulation tissue formation assessed in different groups, comparatively.

Histomorphometry analysis was conducted at 16 days post-treatment to further evaluate the healing process. The assessment was semi-quantitatively by comparative analysis on 5 point scales: 0 (without new epithelialization), 1 (25%), 2 (50%), 3 (75%), and 4 (100%). Moreover, collagen density as informative data was measured and analyzed on the wound site using computer software Image-Pro Plus V.6 (Media Cybernetics, Inc., Silver Spring, USA).

### Statistical analysis

Minitab 17 software (Minitab Inc., State College, USA) was used to analyze the obtained data statistically. The one-way ANOVA was used, followed by the Tukey post hoc test for multiple comparisons. The data were expressed as the mean ± standard deviation (SD). In all evaluations, *P* < 0.05 was considered to be statistically significant.

### Ethical approval

The animal study was conducted on 24 male adult Wistar rats after approval of the ethics committee of Kermanshah University of Medical Sciences. All applicable international, national and institutional guidelines for the care and use of animals were followed.

## Data Availability

The datasets generated during and/or analysed during the current study are available from the corresponding author on reasonable request.
